# How Does the Degree of Valence Influence Affective Auditory P300-Based BCIs?

**DOI:** 10.3389/fnins.2019.00045

**Published:** 2019-02-19

**Authors:** Akinari Onishi, Seiji Nakagawa

**Affiliations:** ^1^Center for Frontier Medical Engineering, Chiba University, Chiba, Japan; ^2^Department of Medical Engineering, Graduate School of Engineering, Chiba University, Chiba, Japan; ^3^University Hospital Med-Tech Link Center, Chiba University, Chiba, Japan

**Keywords:** BCI, BMI, P300, EEG, affective stimulus

## Abstract

A brain-computer interface (BCI) translates brain signals into commands for the control of devices and for communication. BCIs enable persons with disabilities to communicate externally. Positive and negative affective sounds have been introduced to P300-based BCIs; however, how the degree of valence (e.g., very positive or positive) influences the BCI has not been investigated. To further examine the influence of affective sounds in P300-based BCIs, we applied sounds with five degrees of valence to the P300-based BCI. The sound valence ranged from very negative to very positive, as determined by Scheffe's method. The effect of sound valence on the BCI was evaluated by waveform analyses, followed by the evaluation of offline stimulus-wise classification accuracy. As a result, the late component of P300 showed significantly higher point-biserial correlation coefficients in response to very positive and very negative sounds than in response to the other sounds. The offline stimulus-wise classification accuracy was estimated from a region-of-interest. The analysis showed that the very negative sound achieved the highest accuracy and the very positive sound achieved the second highest accuracy, suggesting that the very positive sound and the very negative sound may be required to improve the accuracy.

## 1. Introduction

A brain-computer interface (BCI), also referred to as a brain-machine interface (BMI), translates human brain signals into commands that can be used to control assistive devices or communicate externally with others (Wolpaw et al., 2000, 2002). For instance, BCIs are used to help persons with disabilities interact with the external world (Birbaumer and Cohen, 2007). BCIs decode brain signals obtained by invasive measurements such as electrocorticography or by non-invasive measurements such as scalp electroencephalography (EEG) (Pfurtscheller et al., 2008). One of the most successful BCIs utilizes EEG signals in response to stimuli – i.e., event-related potentials (ERPs). In particular, when a subject discriminates a rarely encountered stimulus from a frequently encountered stimulus, the ERP elicits a positive peak at around 300 ms from stimulus onset; this peak is named the P300 component. ERPs with P300 have been applied to BCIs, and such a system is called a P300-based BCI (Farwell and Donchin, 1988).

Early studies of the P300-based BCI commonly used visual stimuli. For instance, Farwell and Donchin (1988) proposed a visual P300-based BCI speller that intensified letters in a 6 × 6 matrix by row or by column (row-column paradigm). Townsend et al. (2010) proposed an improved P300 speller that highlighted letters in randomized flashing patterns derived from a checkerboard, which was called the checkerboard paradigm. Ikegami et al. (2014) developed a region-based two-step P300 speller that could use the Japanese Hiragana syllable, and took advantage of a green/blue flicker and region selection. In addition, facial images (e.g., famous faces and smiling faces) were applied to visual P300-based BCIs, which resulted in innovative improvement (Kaufmann et al., 2011; Jin et al., 2012, 2014). Although visual P300-based BCIs have been improved, and have shown to be effective in clinical studies, they still depend on the eye condition and can be affected by whether the eyes are open or closed, limited eye gaze, or limited sight. Therefore, it is important that other sensory modalities be studied and improved upon.

In addition to visual stimuli, other modalities, for example auditory stimuli, have been studied. In an early study of auditory P300-based BCIs, auditory stimuli, such as the sound of the word “yes,” “no,” “pass,” and “end” were assessed in a BCI developed by Sellers and Donchin (2006). Klobassa et al. (2009) examined the use of bell, bass, ring, thud, chord, and buzz sounds. Moreover, by pairing numbers with letters using a visual support matrix, Furdea et al. (2009) succeeded in spelling letters by counting audibly pronounced numbers. In addition, beep sounds were used as auditory stimuli and the effects of pitch, duration, and sound source direction were assessed by Halder et al. (2010). Höhne et al. (2010) developed a two-dimensional BCI that varied in sound pitch and location of sound source. Although the auditory BCI is advantageous because it is independent of eye gaze, auditory BCIs have shown worse performance than visual BCIs in several studies (Sellers and Donchin, 2006; Wang et al., 2015), and it is clear that improvements to the auditory BCI are required. Thus, we focused on improving auditory stimuli for BCIs in the current study.

The auditory P300-based BCI can be improved by using sophisticated stimuli. Schreuder et al. (2010) demonstrated that presenting sounds from five speakers in different locations resulted in better BCI performance than that obtained using a single speaker. Additionally, Höhne et al. (2012) evaluated spoken or sung syllables as natural auditory stimuli, and compared to the artificial tones, found that the use of natural stimuli improved the users' ergonomic ratings and the classification performance of the BCI. Simon et al. (2014) also applied natural stimuli of animal sounds. Guo et al. (2010) employed sound involving spatial and gender properties together with discriminating properties of sounds (active mental task), which served to enhance the late positive component (LPC) and N2. Recently, Huang et al. (2018) explored the use of dripping sounds and found that the BCI classification accuracy was higher than when beeping sounds were used. We previously applied sounds with two degrees of valence (positive and negative) to the auditory P300-based BCI (Onishi et al., 2017). We confirmed the enhancement of the late component of P300 in response to those stimuli. However, how degrees of valence (e.g., very positive or positive) influence the P300-based BCI remains unknown.

This study aimed to clarify how degrees of valence in sounds influence the auditory P300-based BCIs. Five sounds with different degrees of valence were applied to the P300-based BCI: very negative, negative, neutral, positive, and very positive. We hypothesized that the valence should exceed a certain degree because the amplitude of P300 was not in proportion to the emotion (Steinbeis et al., 2006). Since the auditory P300 BCI requires a larger amount of training data, and can cause fatigue when applied to the BCI separately, we applied these sounds to the BCI together. The influence caused by the sound valences was then analyzed offline with cross-validation. To confirm the valence of those sounds, the Scheffe's method of paired comparison was applied. We also performed a waveform analysis using the point-biserial correlation coefficient to reveal ERP components that contributed to the classification. Based on the waveform analysis, a region-of-interest (ROI) was identified, and then specially designed cross-validation was performed to estimate offline stimulus-wise classification accuracy. The online performance of the BCI and its preliminary feature extraction process has previously been demonstrated; however, the effect of these affective sounds was not evaluated (Onishi and Nakagawa, 2018). Therefore, in the current study, the influence of affective auditory stimuli on the BCI was evaluated based on the waveform analysis and the stimulus-wise classification accuracy. In contrast to our previous study (Onishi et al., 2017), which revealed whether affective sound is effective to the BCI, the current study aimed to clarify how degrees of valence influence the BCI. The name of the late component seen in our previous study (Onishi et al., 2017) is not consistent within the literature; P300, P3b, P600, late positive component, or late positive complex are used (Finnigan et al., 2002). In the current study, we used the term late component of P300 in reference to those ERP components.

## 2. Methods

### 2.1. Subjects

Eighteen healthy subjects aged 20.6 ± 0.8 (9 females and 9 males) participated in this study. All participants were right-handed as assessed by the Japanese version of the Edinburgh Handedness Inventory (Oldfield, 1971). All subjects gave written informed consent before the experiment. This experiment was approved by the Internal Ethics Committee at Chiba University and conducted in accordance with the approved guidelines.

### 2.2. Stimuli

Five cat sounds, representing five different valences (very negative, negative, neutral, positive, and very positive), were prepared. Sounds were cut to 500 ms, then rises and falls of the sounds were linearly faded in and out. The root-mean-square of each sound was equalized. Detailed conditions of sound processing were presented in Table 1. The sound was emitted through ATH-M20x headphones (Audio-Technica Co., Japan) via audio interface UCA222 (Behringer GmbH, Germany).

**Table 1:** Properties of affective sounds.

**No**.	**Valence label**	**Sound name**	**Source**	**Trimming sample**
1	Very negative	cat-fight	http://taira-komori.jpn.org/	64,910
2	Negative	Cat_Meowing_2- Mr_Smith-780889994	http://soundbible.com/	12,000
3	Neutral	cat6	http://pocket-se.info/	2,769
4	Positive	catvoice	http://taira-komori.jpn.org/	882
5	Very positive	kitty	http://pocket-se.info/	112

Degrees of valence for these sounds were rated and verified using Ura's variation of the Scheffe's method (Scheffé, 1952; Nagasawa, 2002). We used this method instead of visual analog scale because it provides more reliable ratings using the paired comparison of sounds. Specifically, a computer first randomly selected two sounds (sounds A and B) out of the five sounds. Second, a subject listened to sound A, and then sound B, only once. After listening to sounds A and B, the subject rated which sounded more positive by reporting ±3 (+3: B is very positive, 0: neutral, −3: very negative). Participants answered the degrees of valence for a total of 20 sound pairs. The degrees of valence were statistically tested using the analysis of variance (ANOVA). The ANOVA was modeled for Ura's variation of the Scheffe's method, which contains factors of the average of ratings, the individual difference of the ratings, the combination effect, the average of the order effect, and the individual difference of the order effect. Note that the main objective is to reveal the main effect of the averaged ratings, and the others are optional factors. See more detail in (Nagasawa, 2002). The analysis was followed by a comparison of the differences of ratings between each pair using a 99% confidence interval.

### 2.3. EEG Recording

EEG signals were recorded using MEB-2312 (Nihon-Kohden, Japan). EEG electrodes were placed at C3, Cz, C4, P3, Pz, P4, O1, and O2 (Onishi et al., 2017) according to the international 10–20 system. The ground electrode was at the forehead and the reference electrodes were at the mastoids. A hardware bandpass filter (0.1–50 Hz) and a notch filter (50 Hz) were applied by the EEG system. The EEG was digitized using a USB-6341 (National Instruments Inc., USA). The sampling rate of the EEG analysis was 256 Hz. Data acquisition, stimulation, and signal processing were completed using MATLAB (Mathworks Inc., USA).

**Figure 1:**
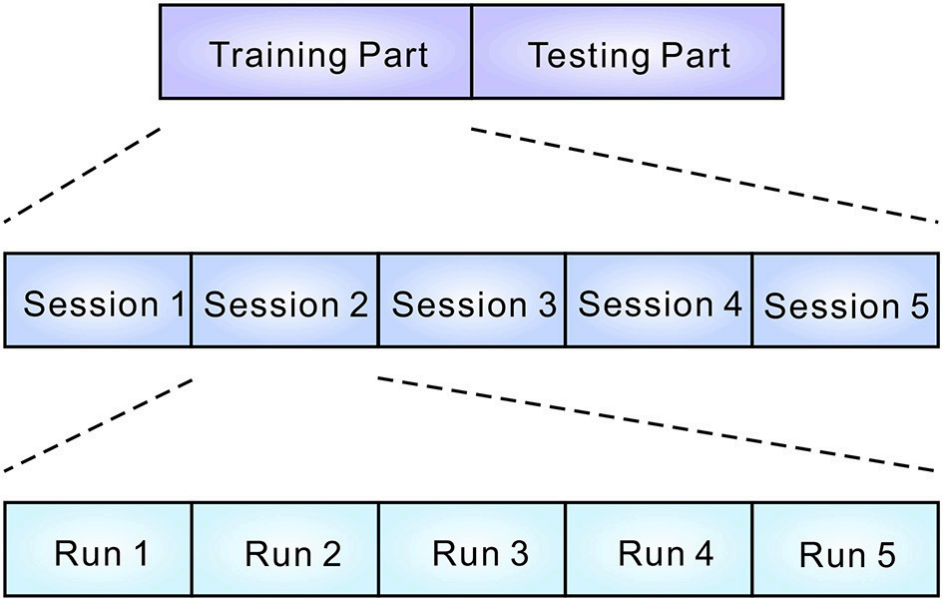
Structure of the experiment. EEG data were recorded during the training and testing parts. Each part consisted of five sessions. In every session, five runs were conducted. Participants were asked to select a sound during a run (see Figure 2).

**Figure 2:**
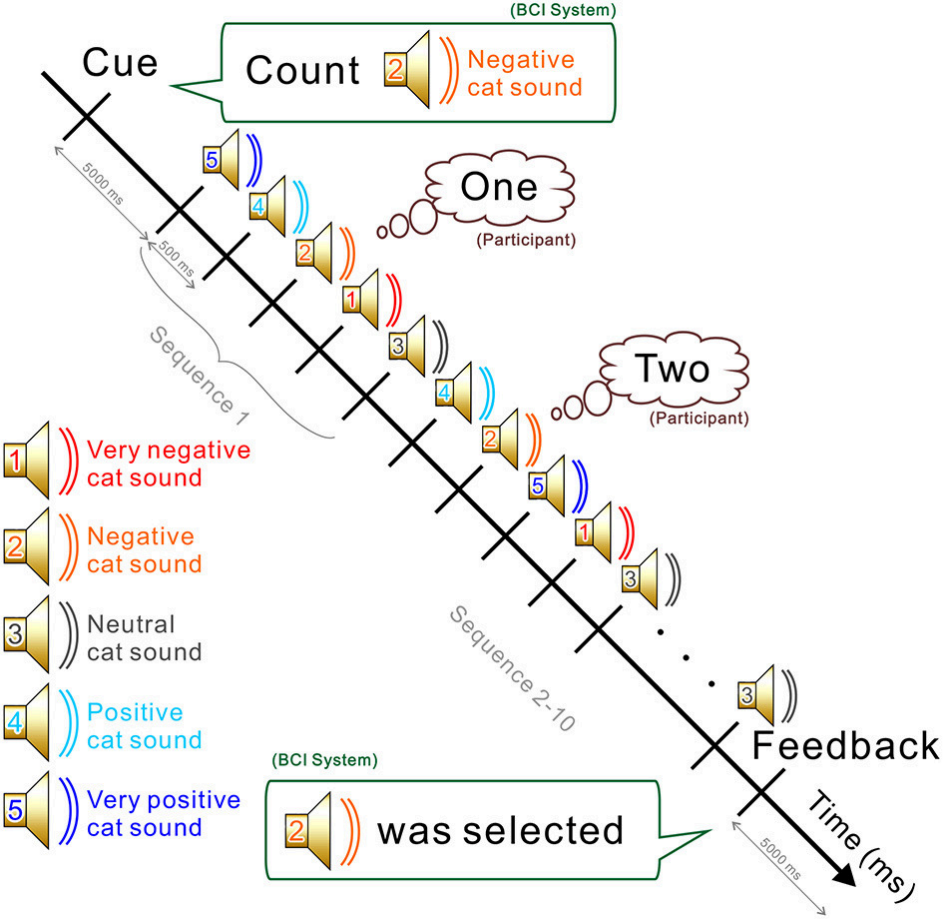
Procedure and the mental task used during a run. The BCI system first asks the participant to count the appearance of a sound (e.g., 2: negative cat sound). All five sounds are then presented in a pseudo-random order. Participants are required to count silently when the designated sound is emitted. At the end of the run, the system estimates which sound was counted based on the EEG signal recorded during the task. The estimated output was fed back to the subject only in the test runs.

The experiment consisted of a training part and a testing part (see Figure 1). Each part contained five sessions. In each session, participants completed five runs. At the beginning of the run, participants were instructed to silently count the number of occurrences of a particular target sound, which was emitted in sequence with other non-target sounds (see Figure 2). Five sounds were then provided in pseudo-random order and each stimulus was repeated 10 times. The stimulus onset asynchrony was 500 ms. EEG signals were recorded during the task. In the testing sessions, outputs were estimated by analyzing EEG signals, and the output was fed back to the subject. For calculating the feedback, smoothing (4 sample window), Savitzky-Golay filter (5th order, 81 sample window), and downsampling (64 Hz) were applied before the classification by the stepwise linear discriminant analysis (SWLDA) (Krusienski et al., 2006). The online classification accuracy was 84.1% (Onishi and Nakagawa, 2018). Between sessions, subjects were asked to take a rest for a few minutes (depending on tiredness). Each sound was selected as a target once in every session. To avoid the effect of tiredness, we applied five sounds together to the BCI, then effects of valences were analyzed offline.

### 2.4. Waveform Analysis

Averaged ERP waveforms for each sound were estimated from the ERP data recorded during the training and testing runs during which a sound was set as the target. The non-target averaged waveforms for each sound were also estimated. All data were preprocessed by applying the baseline removal estimated from –100 to 0 ms waveforms; a software bandpass filter (Butterworth, 6th order, 0.1–20 Hz) was also applied. ERPs that exceeded 80μV were removed.

In order to reveal which ERP components contributed to the EEG classification, the point-biserial correlation coefficients (*r*^2^ values) were estimated (Tate, 1954; Blankertz et al., 2011; Onishi et al., 2017). The point-biserial correlation coefficient is defined as follows:

(1) $$r:=\frac{\sqrt{N_{2}\cdot N_{1}}}{N_{2}+N_{1}}\frac{\mu_{2}-\mu_{1}}{\sigma },$$

where *N*_2_ and *N*_1_ indicate the number of data in target (2) and non-target (1) classes, μ_2_ and μ_1_ are the mean value of target and non-target, and the σ denotes the standard deviation of a sample in a channel. The point-biserial correlation coefficient is equivalent to Pearson correlation between the amplitude of the ERP (continuous measured variable) and classes (dichotomous variable). The squared r (*r*^2^) was used for the waveform analysis. It becomes higher as the difference of mean values between classes is larger and the standard deviation is smaller. We used the method instead of traditional ERP component statistics because it provides rich spatio-temporal information. The *r*^2^ values were evaluated using a test of no correlation and *p* values were corrected using Bonferroni's method. If the *r*^2^ value was not significant, the value was presented as a zero.

### 2.5. Classification

The offline stimulus-wise classification accuracy (Onishi et al., 2017) was computed using stimulus-wise leave-one-out cross-validation (LOOCV). First, training and testing runs, in which a sound was designated as a target, were selected from all 50 runs. Therefore, ten runs were selected in total by the procedure. Second, one run was selected as a testing run and the others as training runs. Third, a supervised classifier was trained on the ERP data during the training runs and then the test data were classified as correct or incorrect. The above two processes were repeated for all 10 runs. The classification accuracy for a sound was calculated as the percentage of correct answers during the 10 runs. We employed this LOOCV to evaluate the influence of each stimulus from the limited amount of data.

We applied baseline removal estimated from –100 to 0 ms waveforms and a software bandpass filter (Butterworth, 6th order, 0.1–20 Hz). They were not downsampled in order to compare the results of waveform analysis and classification. Then they were vectorized before applying classification. We used the SWLDA classifier (Krusienski et al., 2006). In summary, given the weight vector of SWLDA **w** and the preprocessed EEG data of *i*-th stimulus number in *s*-th stimulus sequence (repetition) **x**_*s, i*_, the output î can be estimated as

(2) $$\hat{i}=\underset{i\in I}{\arg\;\max}\sum_{s=1}^{S}w\cdot x_{s,i},$$

where *S*∈{1, 2, …, 10} denotes the maximum number of stimuli used during the offline analysis, and *I*∈{1, 2, …, 5} indicates the list of stimulus numbers (see Table 1). The offline stimulus-wise classification accuracy was calculated *#correctruns*/*#totalruns* fixing *S*. The thresholds of the stepwise method were *p*_in_ = 0.1 and *p*_out_ = 0.15. Training data that exceeded 80μV were removed. Testing data that showed over 80μV amplitude were set to zero to reduce the influence of outliers. The stimulus-wise classification accuracy was statistically tested by the two-way repeated-measures ANOVA, where the factors are the type of stimulus and the number of stimulus sequences (repetitions).

To identify how the components seen in the waveform analysis contributed to the classification, we calculated the stimulus-wise classification accuracy by applying a ROI. The ROI analysis is especially used in fMRI studies to clarify which area of the brain was activated (Brett et al., 2002; Poldrack, 2007). Since ERP studies focus on ERP components spread over channels, spatio-temporal ROI selection was applied in this study. Specifically, the ROI in this study was set to C3, Cz, and C4 in the 400–700 ms. The effect caused by the ROI was the same among comparison conditions. The similar analysis has been applied in P300-based BCI studies to reveal the effect of ERP components (Guo et al., 2010; Brunner et al., 2011). We decided to apply the ROI to reveal the effect of sound valence in response to results of waveform analysis because the automatic feature selection does not ensure which component to select and cannot support the conclusion.

## 3. Results

### 3.1. Subjective Reports

**Figure 3:**
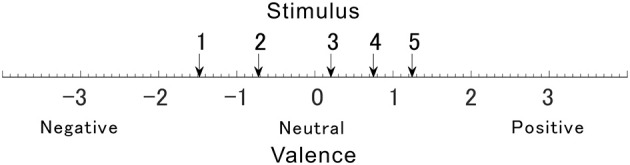
The degree of valence was measured using the Scheffe's method. Arrows numbered 1–5 indicate affective scale values for each sound.

Since the valences of the selected sounds were not confirmed, their valences were verified using Ura's variation of Scheffe's method. Figure 3 shows the valence of each sound. Valences of stimulus 1 to 5 in Table 1 were rated –1.48, –0.72, 0.20, 0.75, and 1.24, respectively. The rated valence was analyzed by the ANOVA. It revealed the significant main effects of the average of ratings, the individual difference of the ratings, and the combination effects (*p* < 0.01). No significant main effect was seen in factors of the average of order effect (*p* = 0.074) and the individual difference of the order effect (*p* = 0.509). Table 2 shows the 99% confidence intervals of valence estimated by Scheffe method. Since any pairs do not contain zero between upper and lower limits, all valence scores were significantly different from each other (*p* < 0.01, see Table 1). The result implies that the valences of the sounds were labeled properly, and they were distributed so that they are different from each other.

**Table 2:** The 99% confidence intervals of valence estimated by Scheffe method.

**Stimulus pair**	**Lower limit**	**Upper limit**
1, 2	–1.00	–0.76
1, 3	–1.93	–1.68
1, 4	–2.47	–2.23
1, 5	–2.97	–2.72
2, 3	–1.17	–0.93
2, 4	–1.72	–1.47
2, 5	–2.21	–1.97
3, 4	–0.79	–0.54
3, 5	–1.28	–1.04
4, 5	–0.74	–0.49

### 3.2. Waveform Analysis

**Figure 4:**
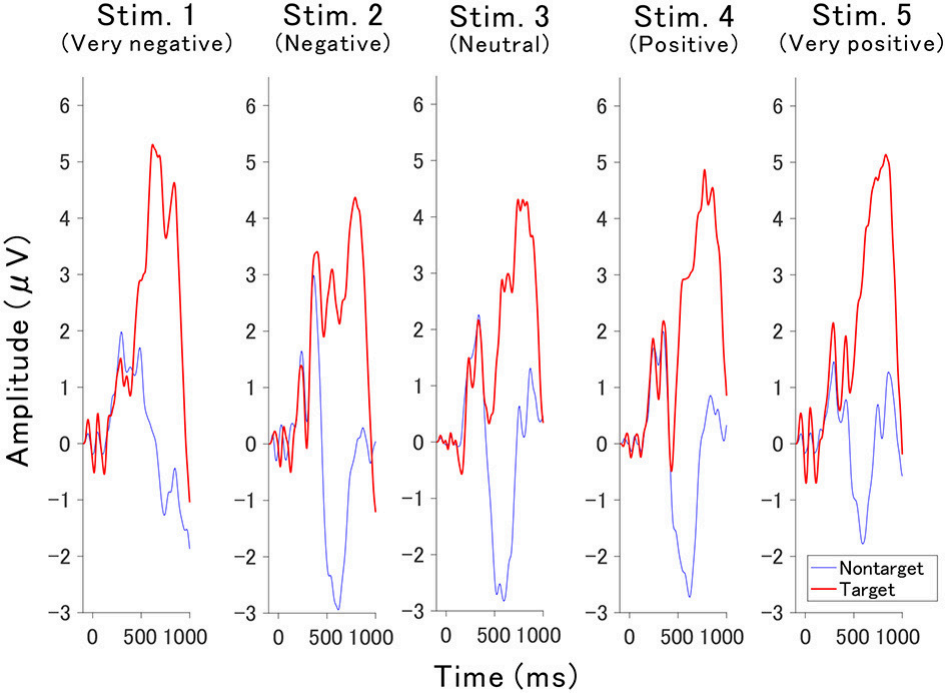
Grand-averaged ERPs for each stimulus recorded from Cz.

The valence of the affective sounds modulated the late component of the P300 amplitude with respect to valence. Figure 4 shows the averaged target and nontarget ERP waveforms obtained at Cz for each sound (waveforms for all channels were also presented in Figures S1–S5). The peak amplitude of the component was lowest in the neutral auditory stimulus (stimulus 3), while the peak was greatest for very negative and very positive auditory stimuli (stimulus 1 and 5, respectively). To know the contribution to the classification and the statistical analysis, the point-biserial correlation coefficient analysis was applied.

**Figure 5:**
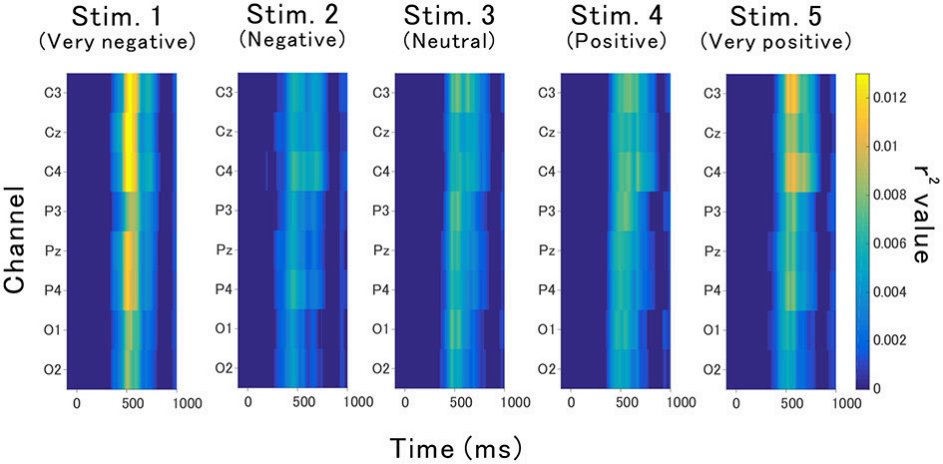
Contribution of each feature to the classification accuracy. This contribution was estimated based on *r*^2^ values.

The point-biserial correlation coefficients (*r*^2^ values) provided in Figure 5 indicate how each auditory stimulus in a channel contributes to the classification. A test of no correlation was applied to each value, then the *r*^2^ values were set to zero if the point-biserial correlation was insignificant. The *r*^2^ values increase around channels C3, Cz, and C4 at approximately 400–700 ms, which corresponds to the late component of P300 (Figure 4).

### 3.3. Stimulus-Wise Classification Accuracy

**Figure 6:**
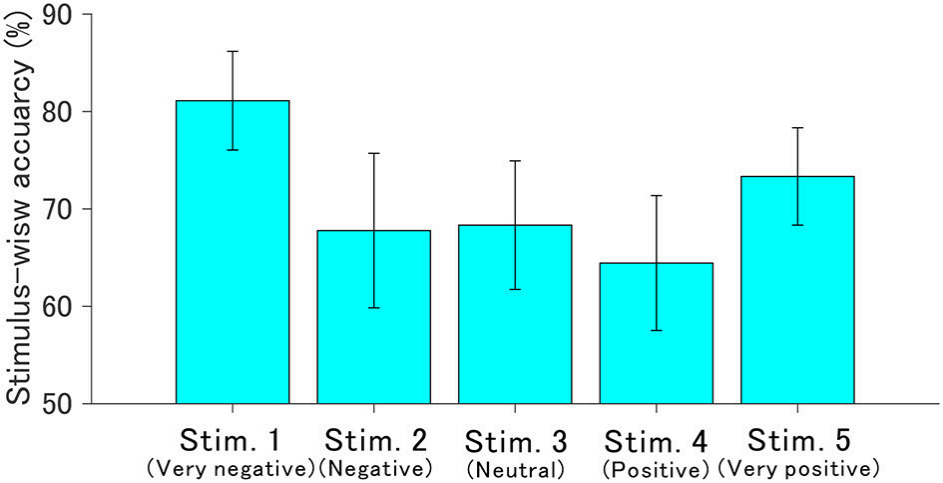
Offline stimulus-wise classification accuracy and standard error for each auditory stimulus. These were calculated based on ERP data in the ROI (400–700 ms in C3, Cz, and C4). The number of stimulus sequences (repetitions) was fixed to 10 times.

**Figure 7:**
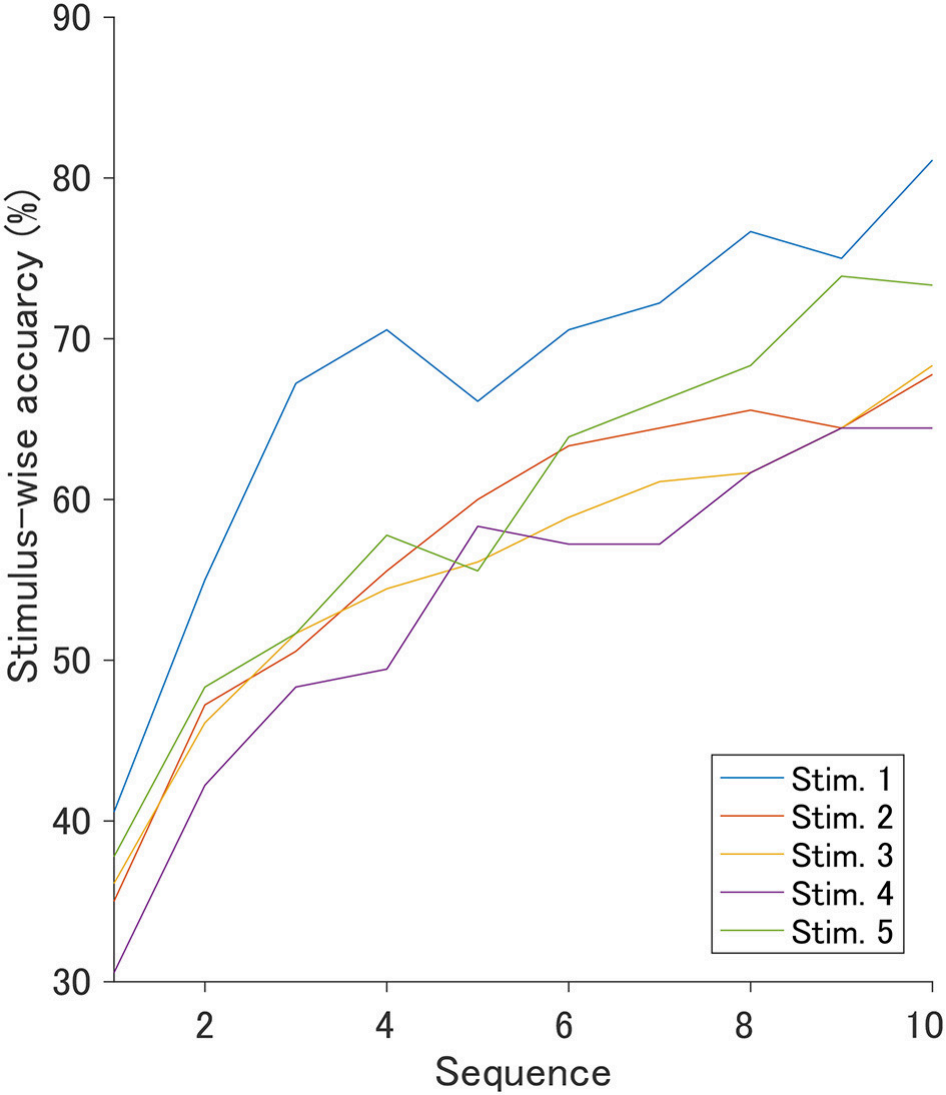
Offline stimulus-wise classification accuracy in 1–10 sequences using a fixed ROI.

The ROI for the classification was set to C3, Cz, and C4 in the 400–700 ms since the *r*^2^ values indicated obvious changes induced by auditory stimuli. Figure 6 shows the stimulus-wise classification accuracy for each sound when the auditory stimuli were each presented 10 times. We found that the very negative sound demonstrated the highest accuracy, the very positive sound demonstrated the second highest accuracy, and the positive sound demonstrated the lowest accuracy. Figure 7 represents the classification accuracy for all 10 stimulus sequences. Two-way repeated-measures ANOVA revealed significant main effects of type of stimulus [*F*_(4, 68)_ = 2.82, *p* < 0.05] and the number of stimulus sequences [*F*_(9, 153)_ = 48.24, *p* < 0.001]. The *post-hoc* pairwise *t*-test revealed that the very negative sound demonstrated the highest accuracy (*p* < 0.001), and the very positive sound showed higher accuracy than the positive sound (*p* < 0.01). In summary, the very negative sound or the very positive sound showed high accuracy.

## 4. Discussion

To clarify how degrees of valence influence the auditory P300-based BCIs, we applied five affective sounds to an auditory P300-based BCI: very negative, negative, neutral, positive, and very positive. Those sounds had significantly different valence from each other. ERP analysis revealed that the very negative and very positive sounds showed high *r*^2^ values or the late components of P300. The very negative sound demonstrated significantly higher stimulus-wise classification accuracy than the other sounds. The very positive sound demonstrated the second highest accuracy, which was significantly higher than the positive sound. These results suggest that highly negative or highly positive affective sounds improve the accuracy. As hypothesized, a certain degree of valence is required to influence the BCI.

Our findings show that the very positive sound improved the stimulus-wise classification accuracy, which is in line with our previous study (Onishi et al., 2017). Additionally, we also demonstrated that the very negative sound improved this accuracy. In our previous study, the negative sound and its control demonstrated similar scores on the affective degree of valence, and therefore, no accuracy difference was found. Considering that these two sounds were scored as negative on the valence scale, the results of the current study is consistent with our previous findings.

This study included only one sound for each valence in order to minimize the variance caused by sounds, and to avoid fatigue. This implies that the results may be sound-specific due to the physical properties of sounds. However, it is unlikely that the effect of affective valence was sound-specific given that the stimuli used in this study were different from those used in our previous study (Onishi et al., 2017). Though different physical properties of sounds were evaluated in those two studies, the results obtained were consistent. Moreover, the P300 amplitude is known to vary with emotional value, which further validates our findings (Johnson, 1988; Kok, 2001).

This study demonstrated that the *r*^2^ values for the late components of P300 displayed by the very positive or very negative sounds were highest around 400–700 ms and centered around the Cz channel. These results were not a simple response to the modality. Wang et al. (2015) examined the *r*^2^ values associated with simple visual gray-white number intensification pronounced number sound, and their combination, and found that the auditory stimuli showed significant *r*^2^ values in fronto-cortical brain regions between 250 and 400 ms, which contradicts our results, while their visual stimuli enhanced the *r*^2^ values mainly around the occipital and parietal brain regions between 300 and 400 ms. Furthermore, they reported that auditory and visual stimuli combined enhanced *r*^2^ values in the large area including parietal area around 300–450 ms. A variety of sounds have been assessed by the point-biserial correlation coefficient. The *r*^2^ values elicited by drums, bass, and keyboard sounds were high within the occipital, parietal, and vertex brain regions between 300 and 600 ms (Treder et al., 2014). Dripping sounds showed Cz channel activity with *r*^2^ values of the P300 (250 to 400 ms) greater than those elicited by beeping sounds (Huang et al., 2018). Japanese vowels, on the other hand, elicited Cz channel activity centered on *r*^2^ values, although different vowels were not presented (Chang et al., 2013). These studies imply that the *r*^2^ values of non-affective auditory BCIs were different from that of affective auditory BCIs. A facial image study demonstrated that upright and inverted facial images resulted in an early peak of *r*^2^ values around 200 ms, although a salient peak was not found for upright facial images (Zhang et al., 2012). A few studies have analyzed affective stimuli using the point-biserial correlation coefficient. One such study analyzed emotional facial images and found large *r*^2^ values at around 400–700 ms, which is also referred to as the late component of P300 or late positive potential (LPP) (Zhao et al., 2011). Affective sounds in our previous study resulted in a late component of P300 at around 300–700 ms; however, this peak was centered around parietal and occipital brain regions (Onishi et al., 2017). These findings suggest that the affective sounds show high *r*^2^ values of the late component of P300. Moreover, the BCI response to affective stimuli may be common amongst modalities given that BCI responses to affective auditory stimuli were similar to those of affective visual stimuli. The effects of multimodal affective stimuli have not been evaluated for use in a BCI, but they would likely elicit ERPs different from those elicited by unimodal stimuli because the brain regions with significant *r*^2^ values were different between different types of stimuli.

As the degree of valence moves away from zero, the *r*^2^ values rapidly increased, implying that the components contributing to classification are not simply in proportion to the valence. A similar tendency was confirmed in previous studies. Steinbeis et al. (2006) evaluated the effect of emotional music on ERPs, focusing on the expectancy violation of chords. The results showed that the amplitude of P300 was enhanced; however, the change was not in proportion to the expectancy. To obtain a late component of P300 with higher *r*^2^ values, the degree of valence may need to exceed a certain threshold.

The stimulus-wise classification accuracy estimated within the ROI was highest when presenting the very positive and the very negative sounds; however, accuracy was lowest when using the positive sound. These results were unexpected given the amplitudes of late component of P300 and *r*^2^ values. The accuracy may not directly reflect the change of peak amplitude of the component or *r*^2^ values since the stepwise method was applied in addition to target and non-target waveform variance.

Due to experimental constraints, we have estimated the stimulus-wise classification accuracy in response to the ROI using LOOCV. Therefore, we should consider the risk of overfitting because a portion of the information in the test data is used for the spatio-temporal feature selection in LOOCV. The offline stimulus-wise classification accuracy in this study was less than the online classification accuracy (84.1%). Thus, the obvious inflation of the accuracy cannot be confirmed. This tendency is in line with similar previous studies (Guo et al., 2010; Brunner et al., 2011). The overfitting is caused easily when the dimension is high (Blankertz et al., 2011). We think that the current analysis is hard to suffer from overfitting because the simple linear classifier (SWLDA) is further simplified by reducing the spatio-temporal selection using ROI. Moreover, the effect of ROI was controlled in the comparison by equally applying it to all compared conditions.

This study employed all five sounds and evaluated specially designed cross-validation in order to reveal the effect of affective sounds with different degrees of valence. This result can be used for the sound selection for the standard auditory P300-based BCI. When designing multi-command auditory P300-based BCI, very positive or very negative sounds should be employed as much as possible to use the enhanced P300 component, while the neutral sounds should be replaced with the very positive or very negative sounds. However, the mutual effects that occur when employing varieties of sounds in the BCI must be clarified in future studies.

In future studies, methods for classifying auditory BCIs will be necessary. In our previous study, we evaluated the ensemble convoluted feature extraction method, which took advantage of the averaged ERPs of each sound (Onishi and Nakagawa, 2018). Recently, an algorithm based on tensor decomposition for auditory P300-based BCI was proposed (Zink et al., 2016). This algorithm does not require subject-specific training, which improves the utility of the BCIs. These approaches may help establish a more reliable affective auditory P300-based BCI.

## 5. Conclusion

To clarify how the degrees of valence influence the auditory P300-based BCIs, five sounds with very negative, negative, neutral, positive, and very positive sounds were applied to the P300-based BCI. The very positive and very positive sounds showed higher point-biserial correlation coefficients of late component of P300. In addition, the stimulus-wise classification accuracy of sounds is high for the very positive and very positive sounds. These results imply that the accuracy is not in proportion to the valence; However, it improved when utilizing very positive or very negative sounds.

## Author Contributions

AO and SN designed the experiment. AO collected the data. AO analyzed the data. AO and SN wrote the manuscript.

### Conflict of Interest Statement

The authors declare that the research was conducted in the absence of any commercial or financial relationships that could be construed as a potential conflict of interest.
