# Supplementary material for: How Does the Degree of Valence Influence Affective Auditory P300-Based BCIs?
Source: Front Neurosci. 2019 Feb 19;13:45. doi: 10.3389/fnins.2019.00045 (PMC6390079; doi:10.3389/fnins.2019.00045)
Supplement: Supplementary file 1 [file Data_Sheet_1.PDF]

Supplementary Material: How does the degree of valence influence affective auditory P300-based BCIs?

1 SUPPLEMENTARY TABLES AND FIGURES

1.1 Figures

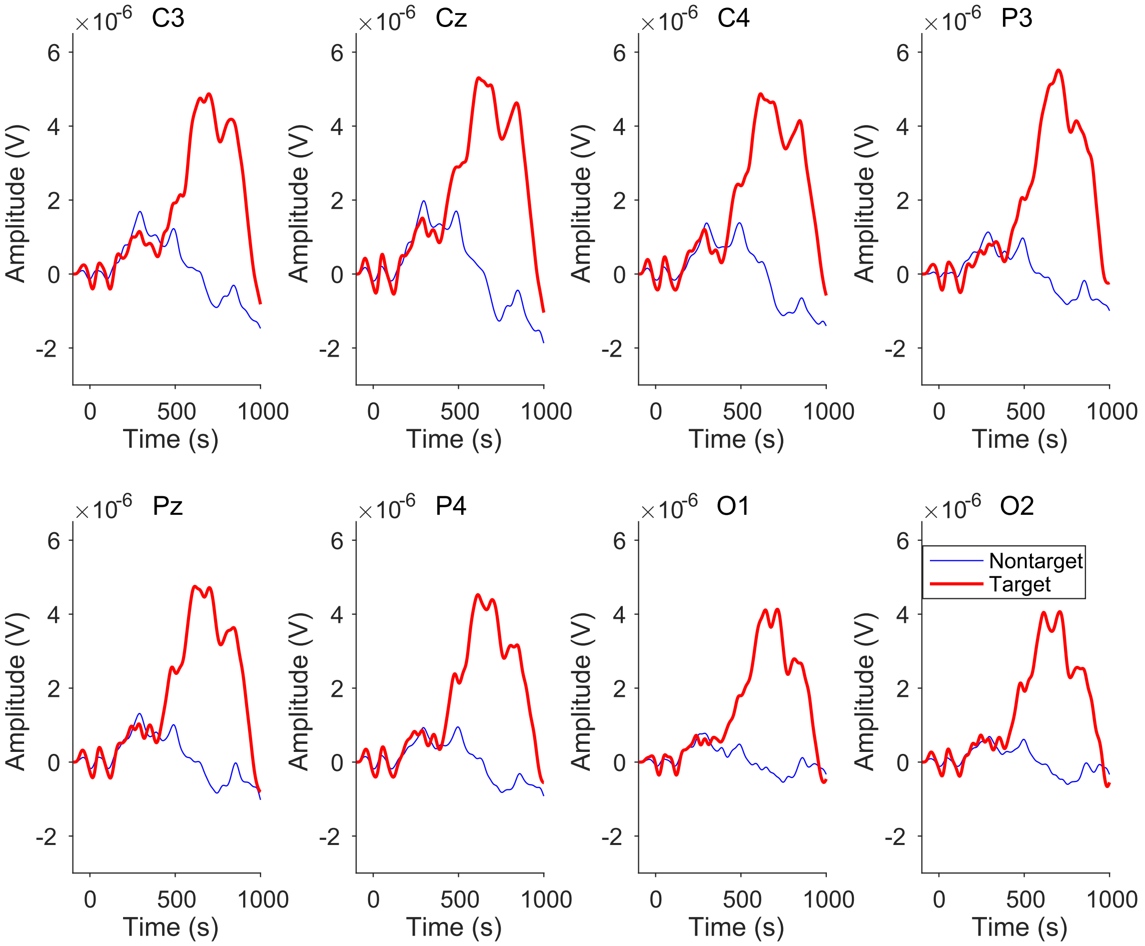

Figure S1. Grand-averaged ERPs for stimulus 1 (very negative) recorded from eight channels.

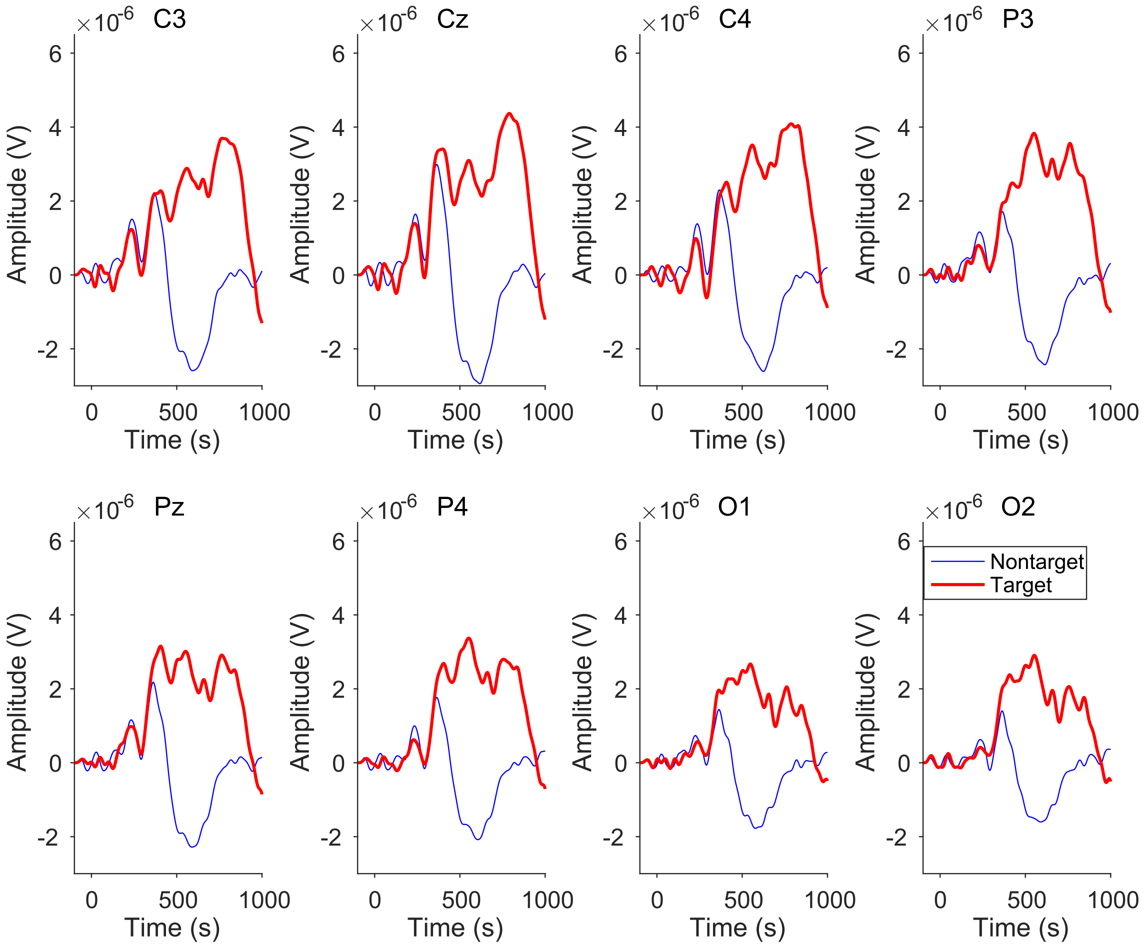

Figure S2. Grand-averaged ERPs for stimulus 2 (negative) recorded from eight channels.

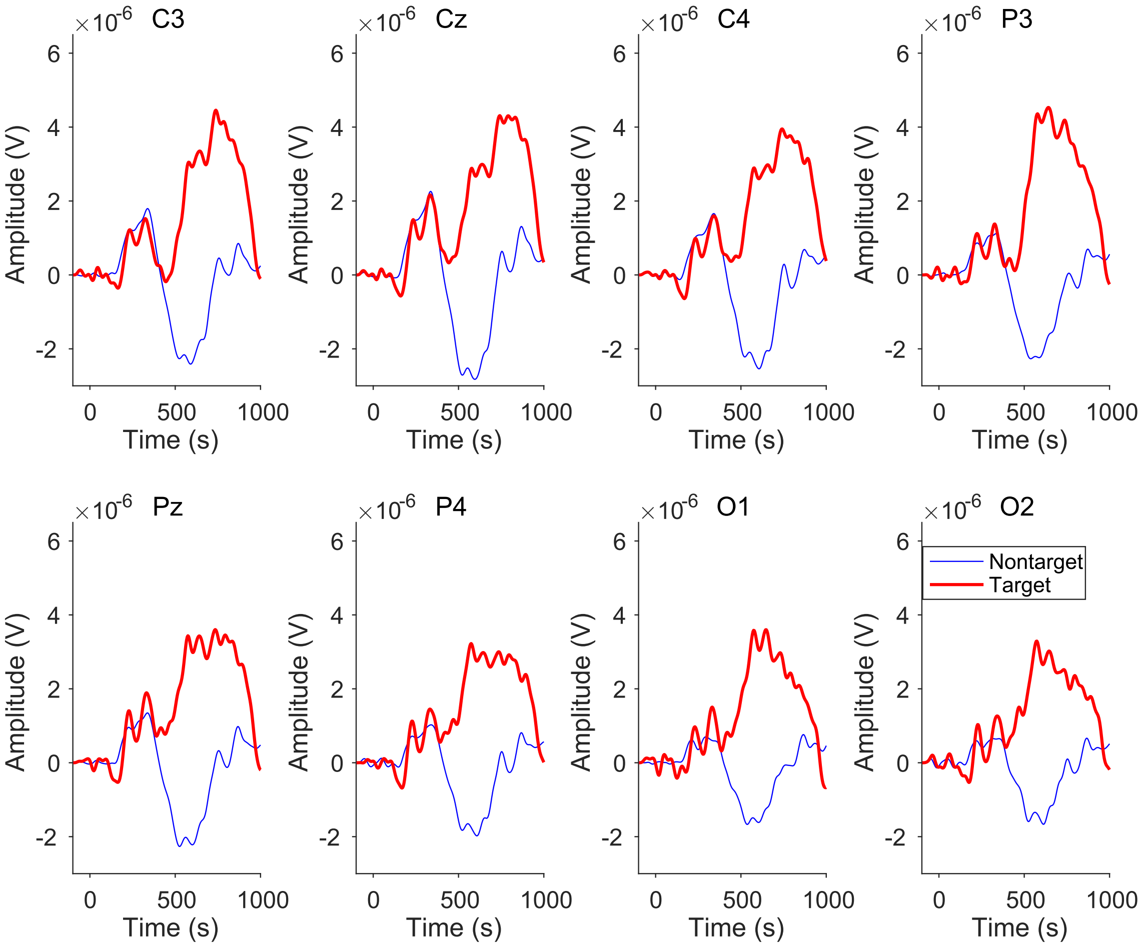

Figure S3. Grand-averaged ERPs for stimulus 3 (neutral) recorded from eight channels.

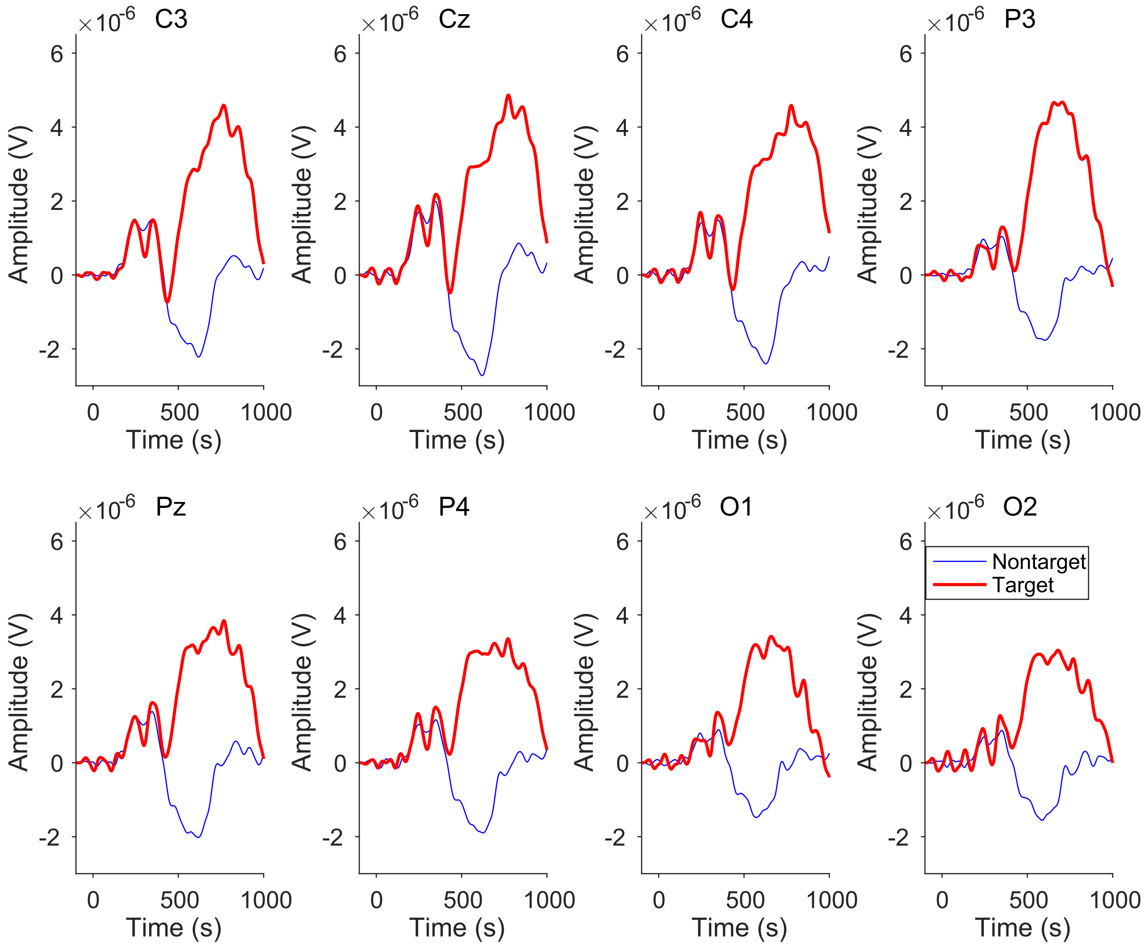

Figure S4. Grand-averaged ERPs for stimulus 4 (positive) recorded from eight channels.

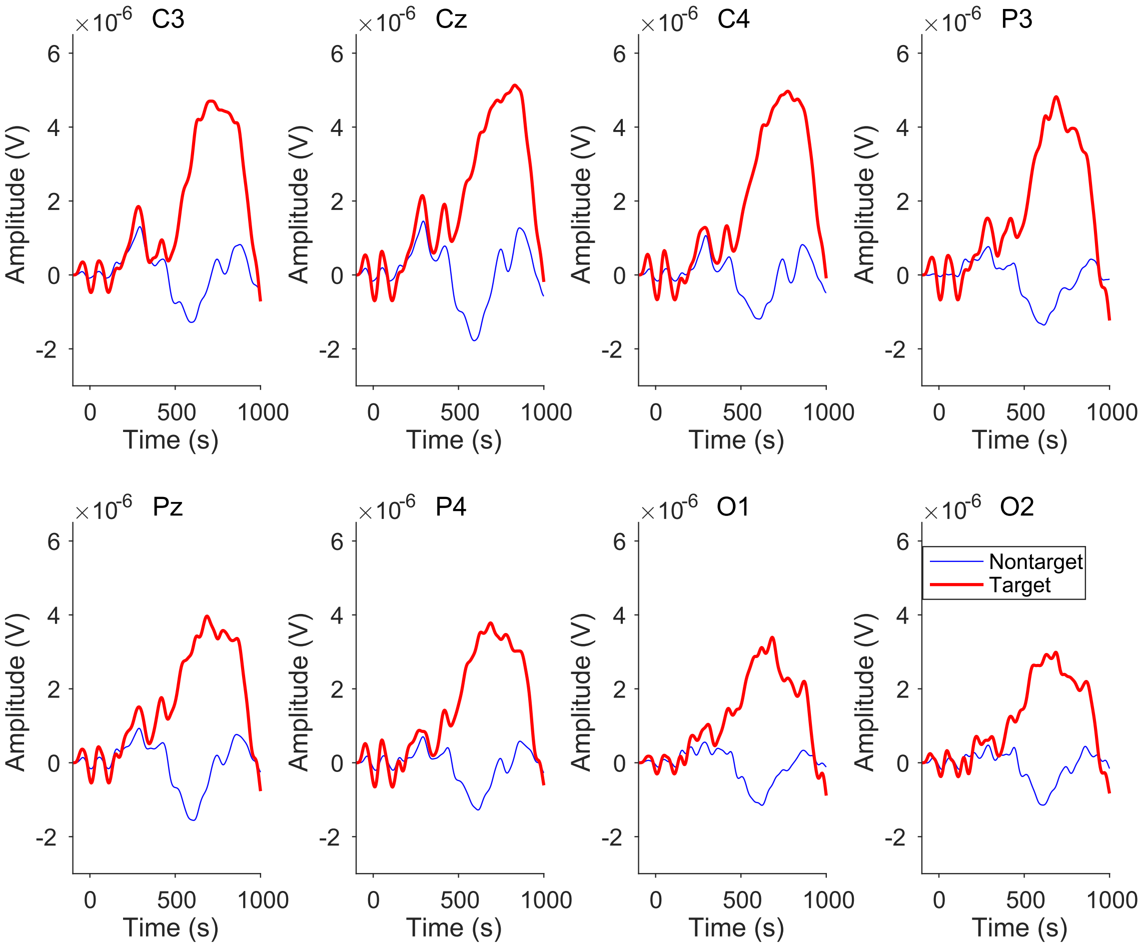

Figure S5. Grand-averaged ERPs for stimulus 5 (very positive) recorded from eight channels.
